# Clinical Performance Evaluation of an Artificial Intelligence-Powered Amyloid Brain PET Quantification Method

**DOI:** 10.1007/s13139-024-00861-6

**Published:** 2024-05-02

**Authors:** Seung Kwan Kang, Mina Heo, Ji Yeon Chung, Daewoon Kim, Seong A. Shin, Hongyoon Choi, Ari Chung, Jung-Min Ha, Hoowon Kim, Jae Sung Lee

**Affiliations:** 1 Brightonix Imaging Inc., Seoul, Korea; 2 Institute of Radiation Medicine, Medical Research Center, Seoul National University College of Medicine, Seoul, Korea; 3 Department of Neurology, College of Medicine, Chosun University and Chosun University Hospital, 365 Pilmun-Daero, Dong-Gu, Gwangju, South Korea; 4 Interdisciplinary Program of Bioengineering, Seoul National University, Seoul, Korea; 5 Artificial Intelligence Institute, Seoul National University, Seoul, Korea; 6 Department of Nuclear Medicine, Seoul National University College of Medicine, 103 Daehak-Ro, Jongno-Gu, Seoul, 03080 Korea; 7 Department of Nuclear Medicine, College of Medicine, Chosun University and Chosun University Hospital, Gwangju, Korea

**Keywords:** Amyloid, Alzheimer dementia, Spatial normalization, Deep learning, Quantification

## Abstract

**Purpose:**

This study assesses the clinical performance of BTXBrain-Amyloid, an artificial intelligence-powered software for quantifying amyloid uptake in brain PET images.

**Methods:**

150 amyloid brain PET images were visually assessed by experts and categorized as negative and positive. Standardized uptake value ratio (SUVR) was calculated with cerebellum grey matter as the reference region, and receiver operating characteristic (ROC) and precision-recall (PR) analysis for BTXBrain-Amyloid were conducted. For comparison, same image processing and analysis was performed using Statistical Parametric Mapping (SPM) program. In addition, to evaluate the spatial normalization (SN) performance, mutual information (MI) between MRI template and spatially normalized PET images was calculated and SPM group analysis was conducted.

**Results:**

Both BTXBrain and SPM methods discriminated between negative and positive groups. However, BTXBrain exhibited lower SUVR standard deviation (0.06 and 0.21 for negative and positive, respectively) than SPM method (0.11 and 0.25). In ROC analysis, BTXBrain had an AUC of 0.979, compared to 0.959 for SPM, while PR curves showed an AUC of 0.983 for BTXBrain and 0.949 for SPM. At the optimal cut-off, the sensitivity and specificity were 0.983 and 0.921 for BTXBrain and 0.917 and 0.921 for SPM12, respectively. MI evaluation also favored BTXBrain (0.848 vs. 0.823), indicating improved SN. In SPM group analysis, BTXBrain exhibited higher sensitivity in detecting basal ganglia differences between negative and positive groups.

**Conclusion:**

BTXBrain-Amyloid outperformed SPM in clinical performance evaluation, also demonstrating superior SN and improved detection of deep brain differences. These results suggest the potential of BTXBrain-Amyloid as a valuable tool for clinical amyloid PET image evaluation.

## Introduction

The role of brain imaging in the diagnosis and treatment of neurodegenerative diseases continues to increase. This is because the brain condition must be evaluated using non-invasive methods due to the nature of brain diseases. Among various techniques for brain imaging, positron emission tomography (PET) visualizes the functional and molecular characteristics of the brain [1]. PET imaging plays an important role in the diagnosis and treatment of various neurodegenerative diseases, such as Alzheimer's dementia and Parkinson's disease, because it provides direct information about the pathophysiology of each patient with neurodegenerative disease [2]. Brain deposition of amyloid beta protein is a pathophysiological factor in the diagnosis of Alzheimer's dementia, the most common neurodegenerative disease. PET radiopharmaceuticals, which non-invasively visualize amyloid beta protein, have been used clinically since the mid-to-late 2010s, making a significant contribution to the diagnosis and treatment of dementia. According to a study analyzing over 16,000 patients at over 100 hospitals in the United States, the diagnosis of Alzheimer's disease in approximately one-third of patients changed depending on the results of amyloid PET [3].

Interpretation of brain images, such as amyloid or dopamine transporter PET, relies on visual interpretation by specialists in an actual clinical environment. However, visual interpretation has limitations, such as inconsistency between readers and lack of objectivity. In fact, the degree of discrepancy in visual interpretation of amyloid PET between experts was reported to be around 0.69–0.80 kappa statistics [4]. On the other hand, quantitative parameters extracted from brain PET images provide objective information about the progression of pathophysiology in various brain regions [5]. In addition, these parameters can play an important role in patient treatment and management when used in conjunction with clinical information. These quantitative parameters are also used for various researches on neurodegenerative diseases and in the clinical trials to develop novel diagnostic and therapeutic agents.

There are several methods that provide more objective and reproducible quantitative parameters from brain PET images. For example, we can also extract quantitative measures of brain PET activity within specific brain regions by overlaying the segmented brain regions from magnetic resonance imaging (MRI) as regions of interest for quantifying PET data. Assessment of statistical significance of PET signal difference at each voxel across the entire brain is another popular method. However, in actual clinical environments, these quantification methods are limitedly used for the following reasons. First, there is no standardized method for quantification. Second, in most cases, other imaging technologies such as magnetic resonance imaging should be used together. Third, a relatively complex procedure for quantification should be performed, which not only requires understanding of image processing technology, but also requires considerable time and effort in the processing.

Spatial normalization of brain PET images into standard stereotactic space is one of the ways allow fast quantification of them [6–12]. Spatial normalization typically involves linear affine transformations and nonlinear transformations of PET images to have the same shape and orientation as a standard template [13]. Regional PET activity concentration can be automatically extracted from spatially normalized images using a predefined atlas in standard space. Because some radiopharmaceuticals for brain PET imaging provide limited morphological information, accurate brain PET spatial normalization often requires matched 3D MRI [5]. However, using MRI requires additional image processing steps such as PET and MRI co-registration. Moreover, MRI is not always available for all patients. Therefore, several approaches using artificial intelligence (AI) techniques have been proposed to achieve spatial normalization of brain PET images without the assistance of a matching MRI [5, 14, 15]. BTXBrain (Brightonix Imaging Inc., Seoul, Korea) software is a brain PET and single-photon emission computed tomography (SPECT) image quantification platform based on an AI-based fast and reliable spatial normalization algorithm.

The purpose of this study was to evaluate the clinical performance of BTXBrain-Amyloid for quantifying amyloid uptake in PET images and compare it with traditional methods using the Statistical Parametric Mapping (SPM) program. We also explored the potential utility of spatial normalization using BTXBrain-Amyloid in the assessment of basal ganglia area.

## Materials and Methods

### Datasets

This study was retrospectively conducted by reviewing the clinical information and brain imaging data of cognitively normal (CN), amnestic mild cognitive impairment (aMCI), non-amnestic mild cognitive impairment (naMCI), Alzheimer's disease (AD), and other neurodegenerative diseases. Demographic information of the subjects is summarized in Table 1. Information that could identify individuals other than demographic or clinical information required for the study was not included, brain imaging data was de-identified and given anonymization numbers. De-identification of the 3D reconstructed image data was performed by defacing the facial area.

**Table 1:** Demographics of the patients

Diagnosis	N	Age	Sex (M/F)	MMSE
CN	50	74.5 ± 5.9	25/25	26.8 ± 2.8
aMCI	39	72.3 ± 7.3	23/16	25.7 ± 2.4
naMCI	11	70.8 ± 5.9	2/9	25.3 ± 2.7
AD	40	74.3 ± 7.5	20/20	19.4 ± 5.8
Others	10	68.7 ± 7.3	5/5	19.3 ± 7.4

*CN* cognitively normal, *aMCI* amnestic MCI, *naMCI* non-amnestic MCI, *AD* Alzheimer’s disease, *Others* diffuse Lewy body disease, frontotemporal dementia, or Parkinson's disease

We analyzed 150 amyloid PET and corresponding 3D T1 MR images. PET imaging utilized two types of PET/CT scanners. All subjects underwent 20-min PET/CT scans 90 min following an intravenous injection of 300 MBq of ^18^F-florbetaben (^18^F-FBB). Out of the 150 subjects, 110 were scanned using the Discovery ST PET/CT (GE Healthcare, US), featuring an axial FOV of 250 mm and 47 slices with 3.3 mm thickness on a 256 × 256 matrix. The remaining 40 subjects were scanned with the Discovery MIDR (GE Healthcare, US), with specifications including axial FOV of 250 mm and 3.75 mm slice thickness on a 256 × 256 matrix. PET data was acquired in 3D acquisition mode and images were reconstructed using an ordered-subset expectation-maximum iterative reconstruction algorithm.

Visual assessments were conducted by two nuclear medicine physicians. The visual analysis focused on the Regional Cortical Tracer Uptake (RCTU) score in various brain regions including the lateral temporal cortex, frontal cortex, parietal cortex, and posterior cingulate cortex/precuneus [16]. A brain PET image was categorized as amyloid-positive if any of these regions had an RCTU score of 2 or higher, indicating minor amyloid load. In cases of discrepancy between readings, a consensus was reached between the two readers for the final interpretation. Two readers showed very good agreement in reading the images and scoring the conventional and modified RCTU variables (weighted kappa ranged from 0.9 to 1).

For MR, T1-weighted 3D MRI was acquired using 3 T MRI (Siemens AVANTO) for co-registration with PET and MRI-based spatial normalization. Retrospective use of patients' images and demographic data and waiver of informed consent were approved by the Institutional Review Board of our institute.

### Evaluation of Spatial Normalization Quality

All PET images were spatially normalized into the Montreal Neurological Institute (MNI) standard space using AI-based and conventional analytical spatial normalization methods. The AI-based spatial normalization was performed using BTXBrain software without the aid of paired MRI. The AI-based amyloid PET spatial normalization algorithm used in BTXBrain program was trained using approximately 1,000 amyloid PET images acquired from various clinical sites, enabling fast PET spatial normalization without the aid of paired MRI. Conventional spatial normalization was performed using SPM version 12 (SPM12) (Wellcome Trust Centre for Neuroimaging). After co-registering each individual’s amyloid PET with the corresponding MRI, the MR image was spatially normalized, and finally the MRI spatial normalization parameters were applied to the PET images using SPM12.

The spatial normalization quality in AI-based BTXBrain and conventional SPM12 was comparatively evaluated using normalized mutual information between spatially normalized PET images and T1 MRI standard template in MNI space. In addition, a voxel-wise group comparison (*t*-test) was performed between amyloid-positive and amyloid-negative groups using the spatially normalized images. For this purpose and clinical performance evaluation of the programs, amyloid PET images were visually assessed by nuclear medicine specialists. According to visual assessment, the images were divided into negative (*n* = 90) and positive (*n* = 60) groups.

### Clinical Performance Evaluation

To evaluate the clinical performance of two spatial normalization methods, regional PET activity concentrations were extracted from the spatially normalized images using Automated Anatomical Labelling (AAL) atlas. We then obtained regional standardized uptake value ratio (SUVR) values for a composite of frontal, precuneus, posterior cingulate cortex, and lateral temporal regions, using the cerebellum grey matter as a reference region.

The amyloid positive/negative classification accuracies of both methods were assessed using receiver operating characteristic (ROC) curve analysis. Additional precision-recall curve (PR curve) analysis was also performed because the data used in this study showed an imbalanced ratio of 90:60 between negative and positive groups.

## Results

**Fig. 1:**
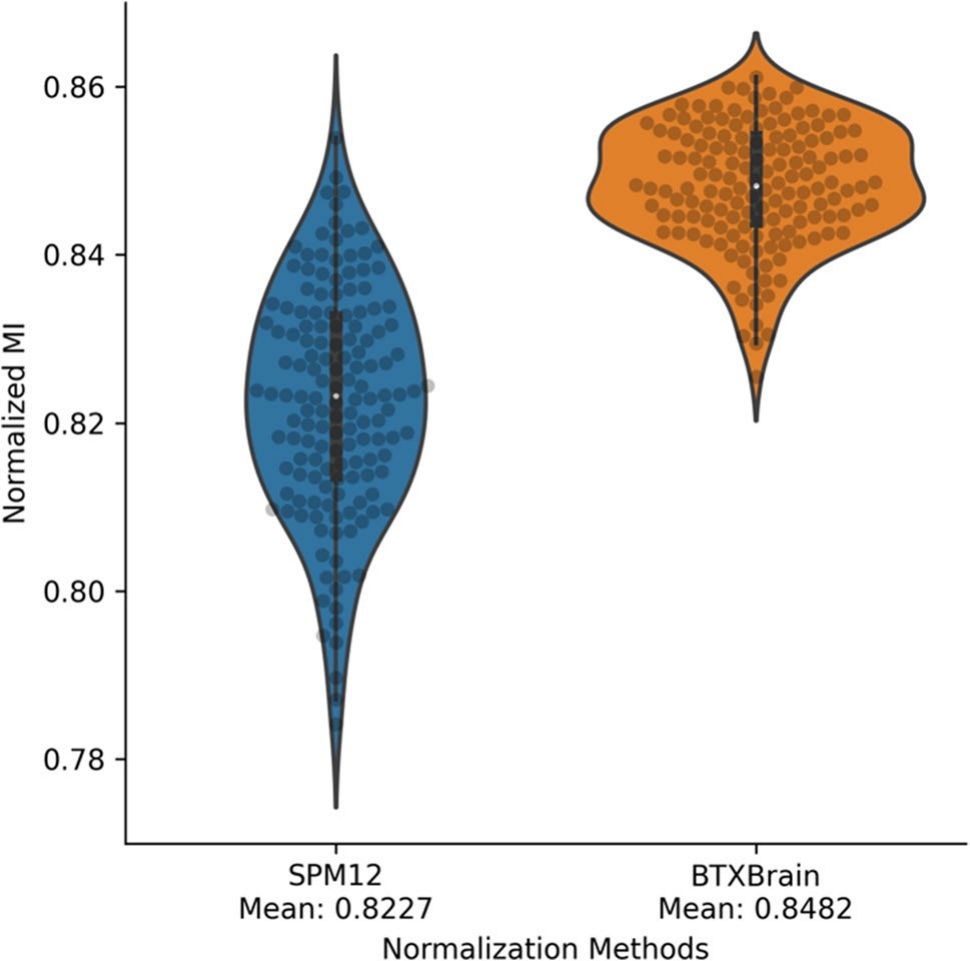
Normalized mutual information, a similarity measure, between spatially normalized PET images and standard T1 MRI template

BTXBrain’s AI-based spatial normalization method using only PET showed higher spatial normalization accuracy than the SPM12 method, which utilized paired MR images. To assess the accuracy of PET spatial normalization, similarity analysis was conducted between the spatially normalized PET images and T1 MRI standard template. The normalized mutual information value indicating similarity to the standard template averaged 0.8482 for BTXBrain and 0.8227 for SPM12 (Fig. 1). Compared to SPM12, BTXBrain exhibited a considerably smaller standard deviation of normalized mutual information 0.0071 For BTXBrain and 0.1339 for SPM12).

**Fig. 2:**
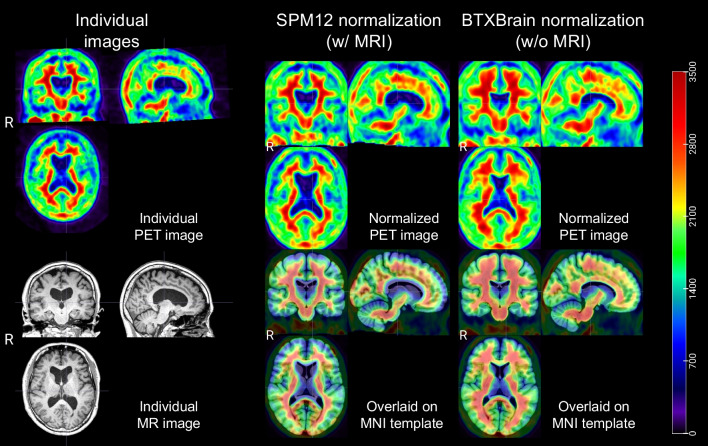
Spatial normalization results of an amyloid negative case (81 y, F)

**Fig. 3:**
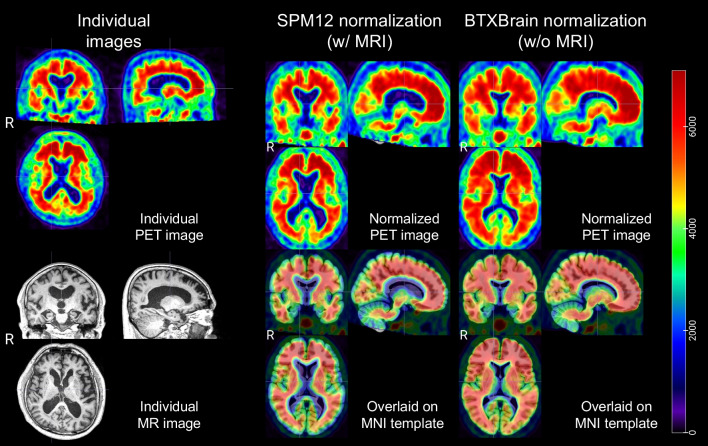
Spatial normalization results of an amyloid positive case (85 y, M)

Figures 2 and 3 show the results of spatial normalization using SPM12 and BTXBrain for reprehensive amyloid negative and positive cases. Only BTXBrain was able to reduce the substantial morphological gaps between individual brain images and standard MNI template, which are caused by hydrocephalus and severe cortical atrophy.

**Fig. 4:**
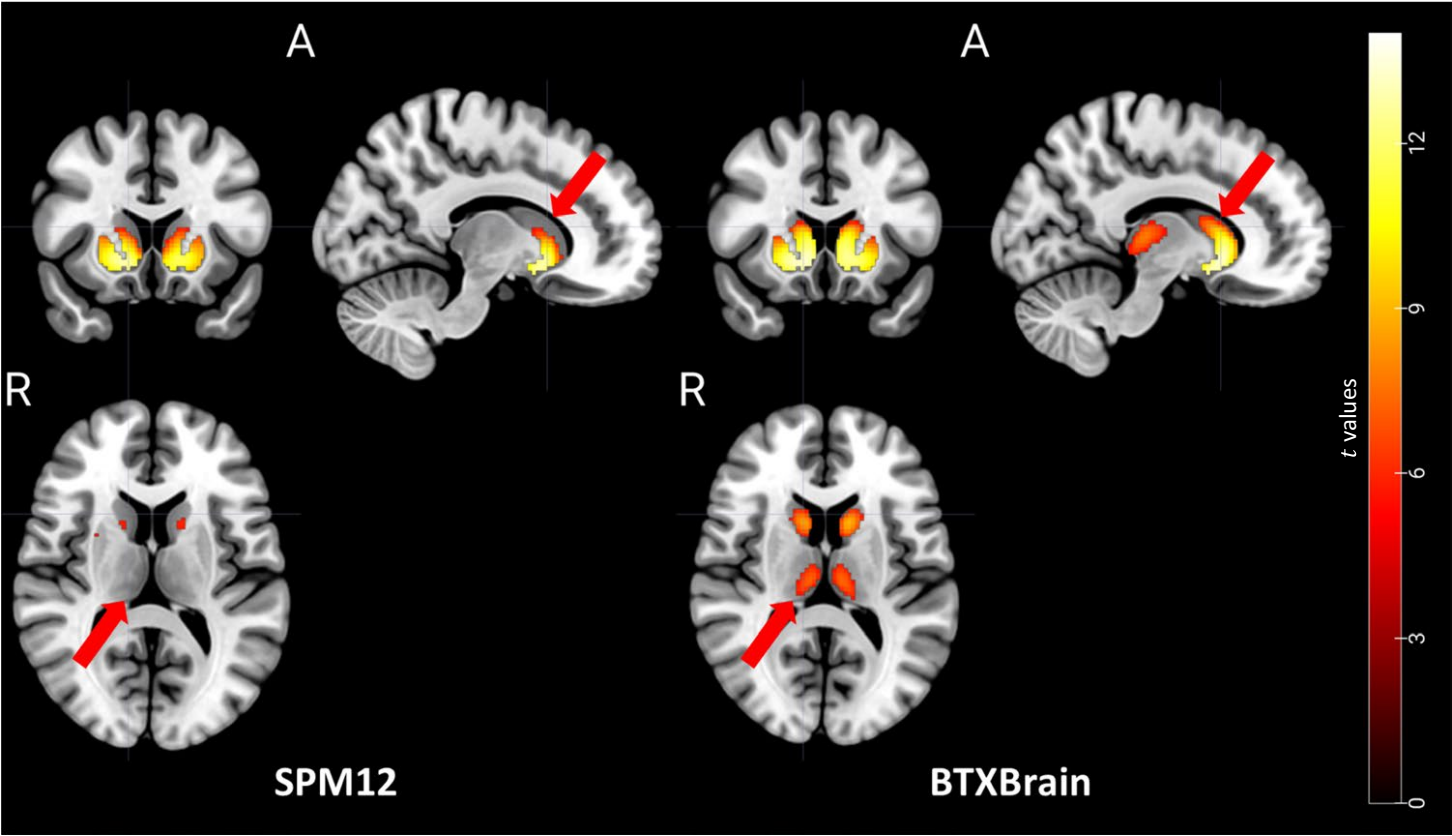
Voxel-wise comparison between amyloid positive and negative groups

To evaluate the impact of differences in spatial normalization accuracy on voxel-level statistical analysis results, voxel-wise SPM analysis (*t*-test) was performed between amyloid-positive and negative groups classified through visual reading by nuclear medicine physicians. When spatially normalized images using BTXBrain were employed, statistically significant differences were observed in the dorsal caudate and thalamus regions, which were not detected when spatial normalization was performed using SPM12 (Fig. 4). The limited accuracy of SPM12 spatial normalization may contribute to high inter-individual variation in PET intensity in deep gray area, consequently reducing the statistical power in estimating inter-group difference. However, BTXBrain, which achieved more accurate spatial normalization in deep brain regions, exhibited the group differences more clearly.

**Fig. 5:**
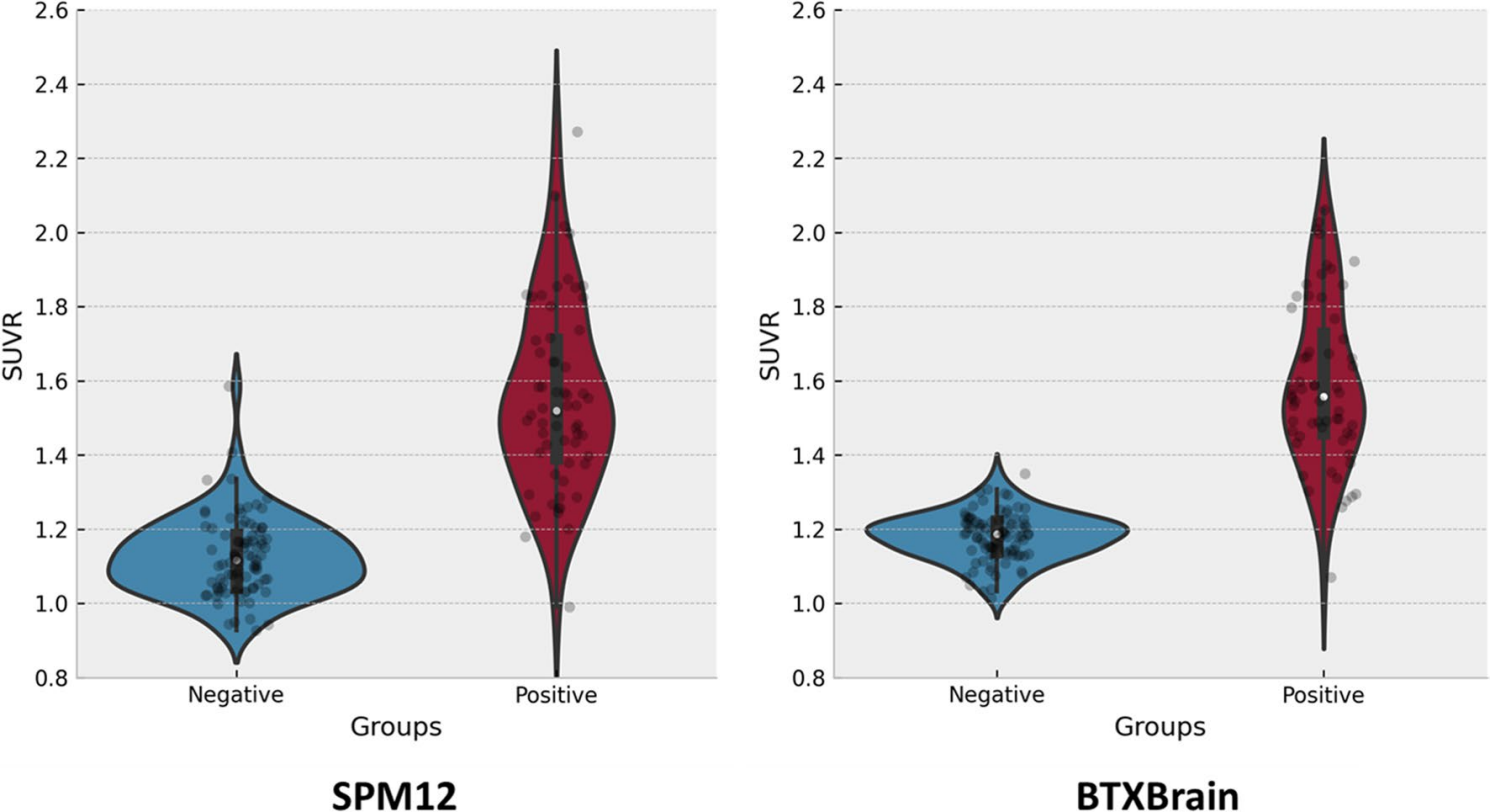
SUVR of amyloid negative and positive groups

**Fig. 6:**
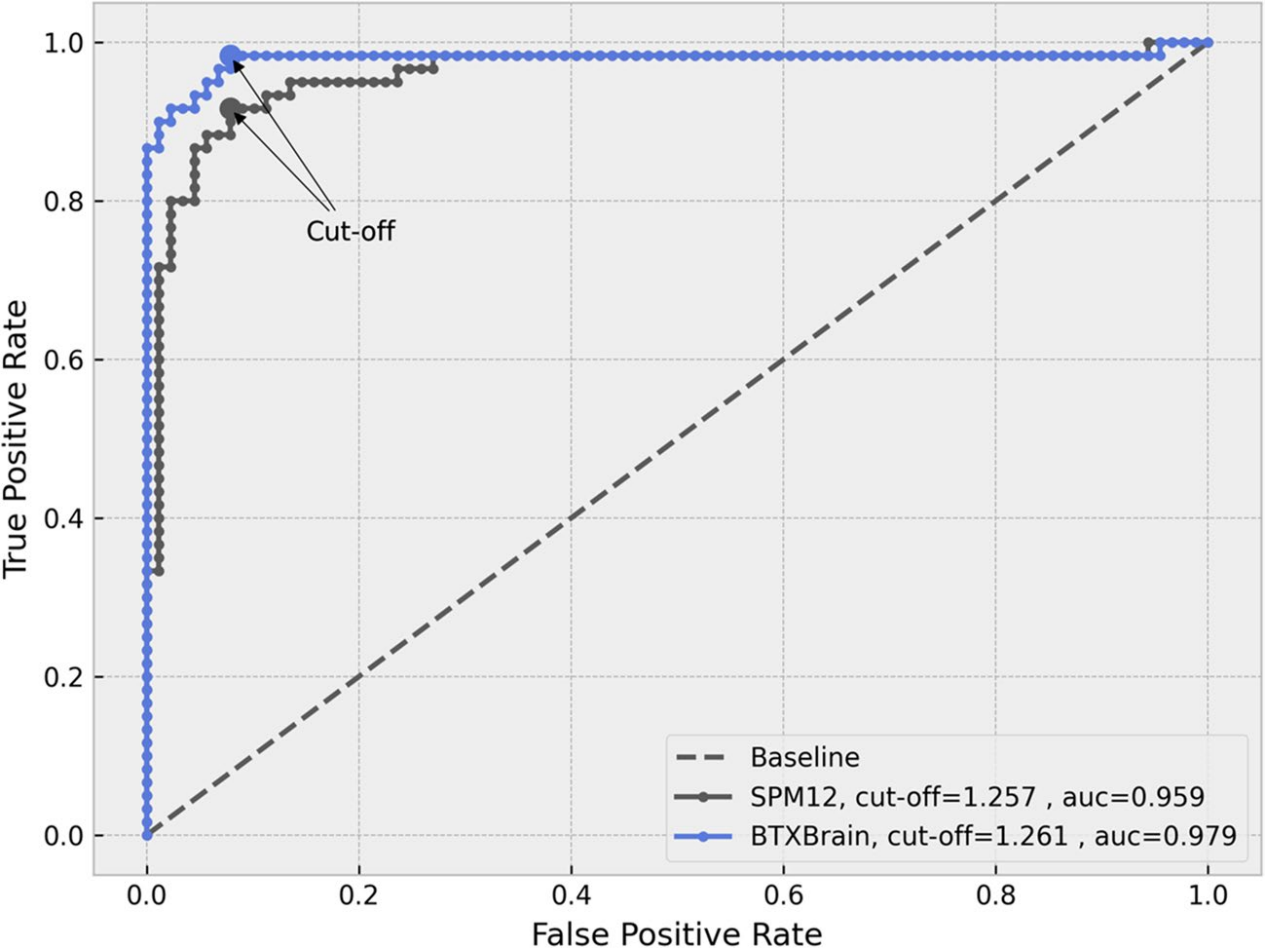
ROC analysis

**Fig. 7:**
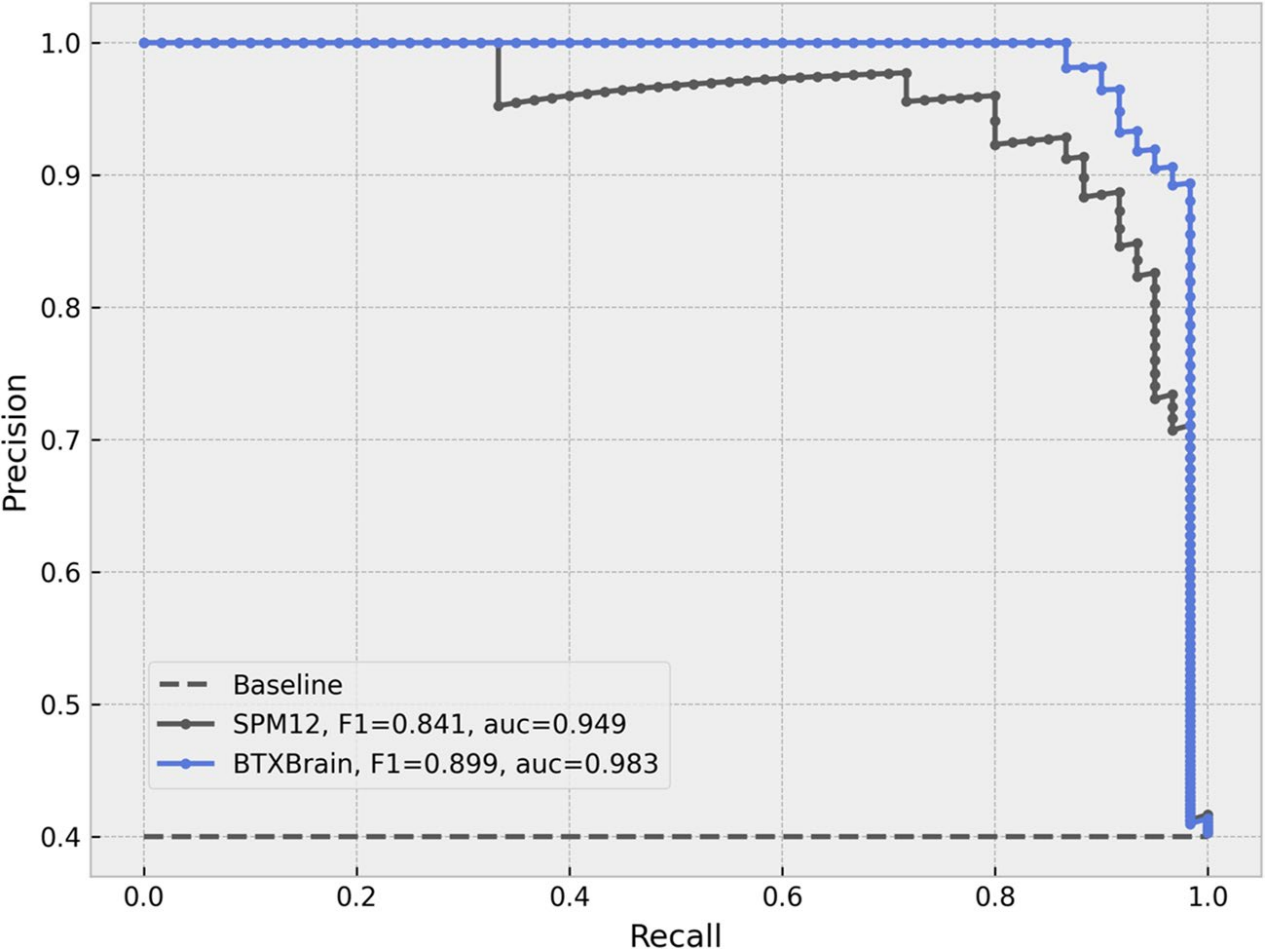
PR analysis

Both BTXBrain and SPM12-based PET quantification methods were able to discriminate between the negative and positive groups (Fig. 5). However, the standard deviation of SUVR was lower in the BTXBrain for both groups (0.06 and 0.21 for negative and positive, respectively) compared to the SPM12 method (0.11 and 0.25 for negative and positive, respectively). In the ROC analysis, the area under the curve (AUC) was 0.979 for BTXBrain and 0.959 for SPM12 (Fig. 6). The sensitivity and specificity at the optimal cut-off were 0.983 and 0.921 for BTXBrain and 0.917 and 0.921 for SPM12, respectively. Precision-recall (PR) curves were also evaluated to account for group imbalances, with an AUC of 0.983 for BTXBrain and 0.949 for SPM12 (Fig. 7). The value of F1 score, which is calculated as the harmonic mean of precision and recall and is known to be useful when data is inhomogeneous, was 0.899 for BTXBrain and 0.841 for SPM12.

PET quantification using SPM12 spatial normalization, including PET and MRI reregistration and regional PET activity calculation, averaged more than 10 min, whereas PET quantification using BTXBrain took only 29 s on average.

## Discussion

The increasing prevalence of neurodegenerative diseases, such as Alzheimer's dementia and Parkinson's disease, has necessitated the development of non-invasive diagnostic and treatment assessment tools, with brain imaging playing a pivotal role in this endeavor. PET has emerged as a valuable imaging technique for visualizing the functional and molecular characteristics of the brain, offering essential insights into the pathophysiology of neurodegenerative diseases. In particular, the ability to visualize amyloid beta protein deposition in Alzheimer’s dementia has significantly contributed to its diagnosis and treatment planning, altering the diagnosis in a substantial proportion of cases based on the results of amyloid PET.

However, the clinical utility of brain PET imaging, including amyloid PET, is hampered by the subjectivity and variability inherent in visual interpretation [17]. This study addresses these limitations by exploring quantitative parameters derived from PET images, which can provide objective information about disease progression and enhance patient management. While several methods for obtaining quantitative parameters from brain PET images exist, they face challenges in standardization, reliance on additional imaging modalities such as MRI, and the complexity of the quantification process.

Spatial normalization of brain PET images, a crucial step in quantification, facilitates the comparison of images across individuals and studies. Spatial normalization also plays a pivotal role in enabling quantitative analysis, as it transforms individual PET images into a common stereotactic space, allowing for objective regional analysis. This study focuses on evaluating the clinical performance of BTXBrain-Amyloid, an AI-based spatial normalization algorithm, and comparing it with traditional methods using SPM.

The results of this study demonstrate the superiority of BTXBrain-Amyloid in spatial normalization accuracy compared to the traditional SPM method, which utilized paired MR images for co-registration. The normalized mutual information values consistently indicated that BTXBrain achieved higher similarity to the standard template, thereby improving the accuracy and reliability of PET spatial normalization. Furthermore, the reduced standard deviation of normalized mutual information in BTXBrain suggests greater consistency and reproducibility compared to SPM, which may be particularly important in multicenter and longitudinal studies. Furthermore, the impact of spatial normalization accuracy on the subsequent voxel-level statistical analysis was evident. When using BTXBrain for spatial normalization, statistically significant differences were observed in regions such as the dorsal caudate and thalamus, which were not detected when employing SPM. This underscores the importance of accurate spatial normalization, particularly in deep brain regions, for improving the sensitivity of detecting group differences and increasing the statistical power of analyses.

The BTXBrain employs a sophisticated approach to nonlinearly register input PET images by utilizing estimated deformation fields to align them with the MNI 152 template. This template, derived from young, healthy individuals (with a mean age of 25.02 and a standard deviation of 4.9), serves as a standardized reference space. Therefore, the deformations may be substantial when input images exhibit significant atrophic regions. To mitigate the potential distortion of images, regularization techniques can be incorporated during training, or the resulting deformation fields can be smoothed. In addition, the image deformation is conducted while preserving the PET pixel intensity which corresponds to radiotracer concentration. Therefore, the quantification results derived from the normalized image using predefined atlases remains accurate. While Kang et al. have demonstrated the robustness of the BTXBrain through internal and external datasets [15], further investigations are warranted to validate its accuracy across diverse image sets and cohorts.

The clinical performance of both BTXBrain and SPM-based PET quantification methods in distinguishing between amyloid-positive and negative groups was assessed through regional SUVR values. Notably, BTXBrain demonstrated lower standard deviations of SUVR for both groups, indicating greater precision in its measurements. ROC curve analysis further highlighted the superior discriminative ability of BTXBrain, with a higher AUC compared to SPM. PR curve analysis accounted for the imbalanced group distribution in this study, further supports the superior performance of BTXBrain.

Beyond improved accuracy and discrimination, BTXBrain offers a substantial advantage in terms of efficiency. The PET quantification process using BTXBrain was remarkably faster, taking only 29 s on average, compared to more than 10 min required by the SPM method, which includes additional steps such as PET and MRI reregistration.

The remarkable performance of BTXBrain-Amyloid in this study illustrates the transformative potential of AI technology in the field of medical imaging. AI-based solutions, like BTXBrain, not only enhance the accuracy, reproducibility, and efficiency of image analysis but also have the potential to revolutionize clinical practice. In the field of medical imaging medical imaging, AI has emerged as a valuable tool, assisting healthcare professionals in tasks ranging from image interpretation and diagnosis to treatment planning and outcome prediction [18–20]. AI can swiftly analyze vast datasets, detect subtle patterns, and provide quantitative insights that were previously unattainable through manual methods [21–27]. As demonstrated in this study, AI-based spatial normalization not only simplifies the quantification process but also improves the sensitivity of detecting disease-related changes, ultimately leading to more precise and personalized patient care.

In summary, this study underscores the clinical potential of BTXBrain-Amyloid as an AI-powered quantification tool for amyloid PET imaging. It not only outperforms traditional methods in terms of accuracy, consistency, and efficiency but also enhances the sensitivity of detecting regional differences in deep brain structures critical for neurodegenerative disease research and diagnosis. The improved precision and discriminative power of BTXBrain in classifying amyloid-positive and negative cases further highlight its clinical relevance in the assessment and management of patients with neurodegenerative diseases. Ultimately, the adoption of such advanced AI-driven tools may revolutionize the field of neuroimaging, aiding in the early diagnosis and effective treatment of these devastating conditions.

## Conclusions

Our study demonstrated that BTXBrain-Amyloid, an AI-based method for quantifying amyloid uptake in brain PET, has superior clinical performance compared to the conventional SPM12 method. In addition, BTXBrain showed improved spatial normalization performance and was more effective in detecting basal ganglia uptake difference in the SPM group analysis. These findings suggest that BTXBrain-Amyloid has the potential to be a useful tool for clinical assessment of amyloid PET images.

## Data Availability

Contact the corresponding authors for data requests. Data will be limitedly available.
